# CO_2_-Responsive Plugging Gel with Sodium Dodecyl Sulfate, Polyethyleneimine, and Silica

**DOI:** 10.3390/polym17060706

**Published:** 2025-03-07

**Authors:** Fanghui Liu, Mingmin Zhang, Huiyu Huang, Rui Cheng, Xin Su

**Affiliations:** 1 Sinopec Key Laboratory of Drilling Completion and Fracturing of Shale Oil and Gas, Beijing 102206, China; liufh.sripe@sinopec.com; 2 Zhejiang Research Institute of Tianjin University, Shaoxing 312369, China; chengrui365@163.com; 3 CNOOC Institute of Chemicals & Advanced Materials (Beijing) Co., Ltd., Beijing 102209, China; huanghy24@cnooc.com.cn; 4 State Key Laboratory of Polymer Materials Engineering, Polymer Research Institute, Sichuan University, Chengdu 610065, China; xinsu@scu.edu.cn

**Keywords:** CO_2_, gel, gas channeling

## Abstract

Gas channeling during CO_2_ flooding poses a significant challenge to enhanced oil recovery (EOR) in heterogeneous reservoirs, limiting both oil recovery and CO_2_ sequestration efficiency. To address this issue, a CO_2_-responsive plugging gel was developed using polyethyleneimine (PEI), sodium dodecyl sulfate (SDS), and nano-silica. The gel formulation, containing 0.8% SDS, 0.8% PEI, and 0.1% nano-silica, demonstrated excellent CO_2_-responsive thickening behavior, achieving a viscosity of over 12,000 mPa·s under selected conditions. The gel exhibited reversible viscosity changes upon CO_2_ and N_2_ injection, shear-thinning and self-healing properties, and stability under high-temperature (90 °C) and high-salinity (up to 20,000 mg/L) conditions. Plugging experiments using artificial cores with gas permeabilities of 100 mD and 500 mD achieved a plugging efficiency exceeding 95%, reducing permeability to below 0.2 mD. These results emphasize the potential of the CO_2_-responsive plugging gel as an efficient approach to reducing gas channeling, boosting oil recovery, and enhancing CO_2_ storage capacity in crude oil reservoirs.

## 1. Introduction

After the completion of primary oil recovery, which relies on the pressure of the reservoir, and secondary oil recovery, which utilizes water injection for displacement, more than two-thirds of the crude oil remains trapped within the reservoir [1,2]. To extract this residual oil, a variety of physical and chemical methods are applied to improve the interactions among oil, gas, water, and rock, which enhances the mobility of crude oil. This procedure, aimed at enhancing the recovery rate, is known as tertiary oil recovery or enhanced oil recovery (EOR) [3,4]. Major techniques include steam flooding, polymer flooding, surfactant flooding, alkaline flooding, and CO_2_ flooding [5,6]. Among these approaches, CO_2_ flooding is considered one of the most efficient techniques. The global implementation of CO_2_ flooding projects has been steadily increasing, establishing it as one of the most widely adopted EOR technologies in the present day [7].

CO_2_-EOR is a technology that can significantly enhance oil recovery. However, pilot tests of CO_2_ injection for oil recovery in certain domestic and international projects have produced suboptimal results, mainly because of severe gas channeling during the CO_2_-EOR process [8,9]. Gas channeling has become a major factor restricting the expansion of the swept area and hindering further improvements in oil recovery during CO_2_-EOR. The gas channeling phenomenon is caused by marked physical differences between CO_2_ and crude oil, as well as reservoir heterogeneity. These differences can be categorized into two main scenarios [10,11,12]: (1) The first is viscous fingering. Under reservoir conditions, the viscosity of CO_2_ (0.03–0.10 mPa·s) [13] is significantly lower than that of crude oil (0.6–10 mPa·s for conventional oil) [12]. This unfavorable mobility ratio leads to the uneven advancement of the CO_2_ slug during displacement. When the CO_2_ flow rate in certain regions becomes too high, it creates finger-like pathways through the crude oil, leading to viscous fingering. This causes instability at the displacement front, premature gas breakthrough, and reduced efficiency in gas utilization. (2) The second is gravity segregation. During CO_2_ flooding, the low density of CO_2_ (0.05–0.5 g·cm^−3^) causes it to gradually migrate above the crude oil, eventually forming gas channels at the top of the flow paths. This leaves a substantial portion of crude oil unswept at the bottom of the reservoir. Gas channeling not only reduces oil recovery but also negatively affects the effectiveness of CO_2_ storage, significantly decreasing the CO_2_ sequestration rate [14,15,16]. Therefore, resolving the gas channeling issue is now among the most critical and urgent challenges in CO_2_ flooding for heterogeneous reservoirs.

Under geological conditions characterized by large-scale heterogeneity (e.g., fractures), the use of gels is often considered the most effective method for mitigating gas channeling during the CO_2_-EOR process [17,18,19]. Gel plugging technology is designed to block high-permeability channels or fractures in the reservoir and to adjust the gas injection profile. This reduces reservoir heterogeneity and forces subsequently injected fluids to move towards low-permeability regions, which enables effective displacement of residual oil [17]. Among polymer/small-molecule hybrid mixtures, those capable of forming pseudo-hydrophobic associative structures demonstrate excellent CO_2_-responsive thickening effects. However, in existing studies, the polymer component often requires laboratory synthesis, limiting the practical advantages of polymer/small-molecule hybrid mixtures. In previous studies [20], a commercially available polymer, polyethyleneimine (PEI), was identified as the polymer component, while sodium dodecyl sulfate (SDS), a common surfactant, served as the small-molecule component. Together, they form a CO_2_-responsive hybrid mixture that creates pseudo-hydrophobic associative structures for thickening. As a result, a CO_2_-responsive thickening polymer/small-molecule hybrid mixture composed of PEI and SDS was developed. Upon CO_2_ stimulation, the hybrid mixture produces pseudo-hydrophobic associative structures, resulting in a thickening effect.

The objective of this study was to develop a CO_2_-responsive plugging gel that utilizes PEI and the anionic surfactant SDS as the main components, along with the introduction of silica nanoparticles. Beginning with the development of CO_2_ flooding gas channeling prevention and plugging technology for low-permeability reservoirs, a CO_2_-responsive plugging gel formulation was developed.

## 2. Materials and Methods

### 2.1. Materials

Polyethyleneimine (PEI, molecular weight = 70,000 g/mol, 30% aqueous solution) and sodium dodecyl sulfate (SDS) were obtained from Macklin Biochemical Co., Ltd. (Shanghai, China). Amino-functionalized nano-silica (diameter, 100 nm) was purchased from MK Nano (Nanjing, China). Carbon dioxide (CO_2_, purity ≥ 99.998%) and nitrogen (N_2_, purity 99.998%) were used without further purification. Throughout the study, deionized water with a resistivity of 18.25 MΩ·cm was provided by a CDUPT-III ultrapure water purifier (Chengdu Ultrapure Technology Co., Ltd., Chengdu, China).

### 2.2. Preparation of the Surfactant Solution

SDS, PEI, and nano-silica were weighed and added to a beaker and then dissolved by stirring in a saline solution.

### 2.3. Viscosity Measurement

Viscosity measurements were conducted using a Physica MCR 302 rotational rheometer (Anton Paar, Graz, Austria). The tests at 25 °C under normal temperature and pressure conditions utilized a CC27 cylindrical rotor, while the temperature sweep tests under high-temperature and high-pressure conditions employed a CC33.2 cylindrical rotor, with a heating rate of 2 °C/min. Prior to testing, both the motor and the test solution were calibrated in sequence. Samples were poured into the sample cell and allowed to stand for 5 min before measurement.

### 2.4. Interfacial and Surface Tension Measurement

The plugging experiments were conducted using a DQ-IV multifunctional polymer flooding apparatus provided by Jiangsu Huaan Scientific Instrument Co., Ltd. (Haian, China) and a ZB-IV vacuum pressure saturation device manufactured by Changzhou Easy Instrument Technology Co., Ltd. (Changzhou, China). The performance of CO_2_ plugging within the polymer was evaluated under simulated reservoir conditions. Prior to the plugging test, the vacuum-dried core’s dimensions and mass were measured. The core was subsequently saturated with brine under vacuum conditions to calculate the pore volume (PV).

The brine-saturated core was placed inside the core holder, where a back pressure of 8 MPa and a confining pressure of 30 MPa were applied. The back pressure of 8 MPa ensured that CO_2_ remained in its supercritical state [21], enabling accurate constant-flow injection. CO_2_ was injected at a flow rate of 2 mL⸱min^−1^, and the pressure difference was continuously monitored. The breakthrough pressure was recorded when CO_2_ penetrated the brine-saturated core. Following this, the test solution was injected into the core at a flow rate of 0.8 mL⸱min^−1^ for 4 h to achieve full saturation. Afterward, CO_2_ was re-injected at a flow rate of 2 mL⸱min^−1^, with real-time monitoring of the pressure difference. The breakthrough pressure was noted when CO_2_ passed through the core saturated with the test solution, marking the conclusion of one plugging experiment. Darcy’s law [10,22] was employed to calculate the core permeability during the plugging process, facilitating a comparative assessment of plugging efficiency across different tests.

## 3. Results

**Figure 1:**
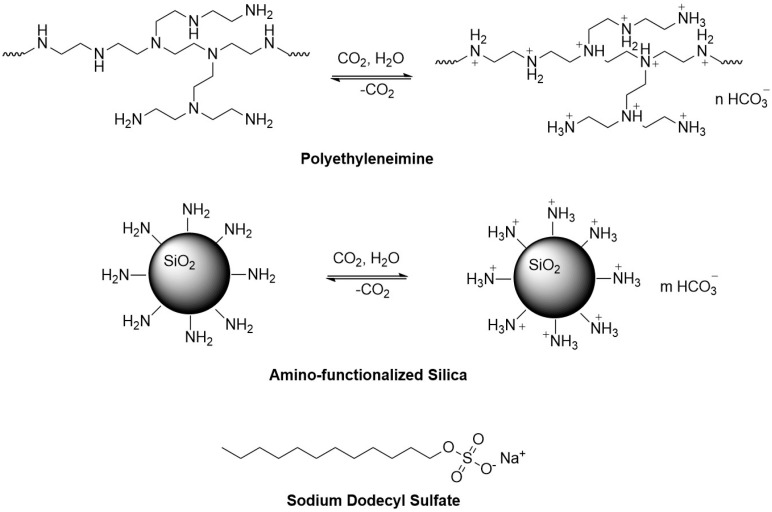
Molecular structures of polyethyleneimine, amino-functionalized nano-silica, and sodium dodecyl sulfate; the CO_2_ switching mechanism of the first two materials.

CO_2_, recognized as an environmentally friendly, low-toxicity, and biocompatible gas, functions as both a displacement medium and a stimulation factor in oil recovery. CO_2_-responsive plugging gels have recently gained attention as an emerging trend. It is found that nanomaterials interact with pseudo-hydrophobic associative polymers and surfactants via chemical adsorption and electrostatic interactions, forming three-dimensional network structures [20]. These structures significantly enhance the viscoelasticity of the solution while reducing fluid loss. Building on this foundation, the present study employs PEI and SDS as primary agents, incorporating amino-functionalized nano-silica to develop a CO_2_-responsive plugging gel (Figure 1). The selection of the three gel chemistries was based on their established performance in forming CO_2_-responsive pseudo-hydrophobic associative structures. The combination of PEI and SDS demonstrates excellent thickening effects under CO_2_ stimulation, providing a logical foundation for this study. SiO_2_ was selected as the nanoparticle additive because of its chemical stability, compatibility with high-temperature and high-salinity reservoir conditions, and ability to enhance the gel’s viscoelasticity through the formation of three-dimensional network structures via chemical adsorption and electrostatic interactions. Experimental results confirmed that the addition of SiO_2_ significantly improved the viscosity and plugging performance of the gel. Other nanoparticles were not evaluated in this study because of resource limitations and the focus on researching a simple and practical formulation. Investigating alternative nanoparticles remains a potential avenue for future research.

### 3.1. Effect of Nano-Silica on Viscosity

**Figure 2:**
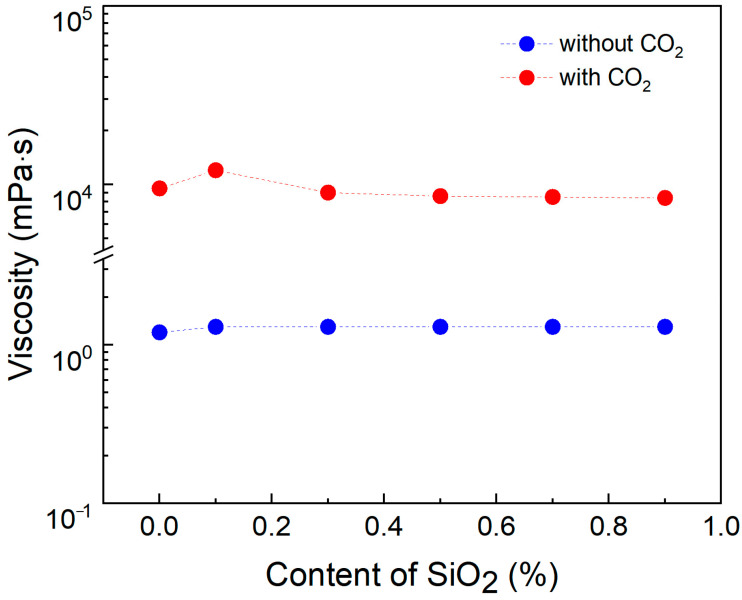
The effect of nano-silica content on viscosity was studied under conditions of 90 °C, a salinity of 5000 mg/L, and a shear rate of 10 s^−1^. The blue points indicate the viscosity measured after CO_2_ injection, whereas the red points represent the viscosity measured before CO_2_ injection.

A solution containing 0.8% SDS and 0.8% PEI was prepared, and varying concentrations of nano-silica (0%, 0.1%, 0.3%, 0.5%, 0.7%, and 0.9%) were added. Subsequently, CO_2_ was injected to measure the viscosity of each solution after the CO_2_ response. The experimental conditions were maintained at 90 °C (the reservoir temperature of Shengli Oilfield) with a solution salinity of 5000 mg/L. As shown in Figure 2 and Figure S1 and S2, adding a small amount of nano-silica increases the viscosity of the solution. However, increasing the concentration of nano-silica beyond a certain level exhibits a negative correlation with viscosity. This suggests that the effectiveness of nano-silica in enhancing viscosity is concentration-dependent, and excessively high concentrations may hinder this enhancement. Adding 0.1% nano-silica increased the viscosity to 12,050 mPa·s. Nano-silica in wormlike micelle solutions can be compared to crosslinking points in rubber elastomer materials. An appropriate number of crosslinking points can enhance the elasticity of rubber, but excessive crosslinking points or crosslinking density can significantly reduce the material’s performance. As for why the addition of 0.1% nano-silica yields the best performance, further research is needed to confirm this. Therefore, the selected formulation for enhancing the viscosity of pseudo-hydrophobic associative polymers was determined to be 0.8% SDS, 0.8% PEI, and 0.1% nano-silica.

### 3.2. Effect of Time for Injecting CO_2_

To further evaluate the viscosity behavior of the SDS–PEI–nano-silica solution during CO_2_ injection, its gelation time was measured using a viscometer. Three solutions were prepared with mass fractions of 0.8% SDS–0.8% PEI–0.1% nano-silica, 0.6% SDS–0.6% PEI–0.1% nano-silica, and 0.4% SDS–0.4% PEI–0.1% nano-silica. At room temperature, CO_2_ was continuously injected into the solutions at a rate of 0.1 L·min^−1^ for 20 min. During this process, the viscosity was measured every 2 min to observe the relationship between injection time and viscosity changes.

**Figure 3:**
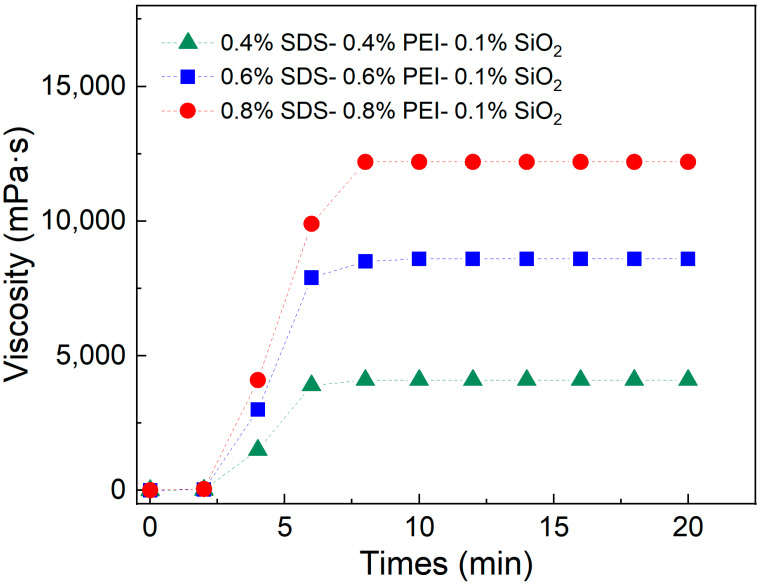
Effect of injecting time on viscosity at a temperature of 90 °C, salinity of 5000 mg/L, and a shear rate of 10 s^−1^.

The viscosity of the SDS–PEI–nano-silica mixture with different mass fractions exhibited distinct phases of change during continuous CO_2_ injection. As shown in Figure 3, CO_2_ began reacting with the solution within the first 2 min. However, the short reaction time resulted in a low degree of protonation of PEI, leading to fewer self-assembled aggregates and insignificant changes in viscosity. When the CO_2_ injection time reached 6 min, the reaction intensified, increasing the number of protonated tertiary amines. This enhanced the binding ability of SDS with the protonated amines, leading to a gradual increase in viscosity. The viscosity reached its maximum after 8 min of CO_2_ injection. Beyond this point, the viscosity remained stable despite continued CO_2_ injection, indicating that the reaction in the solution had reached saturation. At this stage, PEI was fully protonated, and the number of pseudo-hydrophobic associative polymers formed through self-assembly reached its maximum. Thus, the mixture required approximately 8 min to transition from initial CO_2_ contact to complete gelation.

### 3.3. Reversibility Test

In the study of CO_2_-responsive gels, the solution transitions from a low-viscosity state to a high-viscosity state upon exposure to CO_2_. When an inert gas (N_2_) is subsequently introduced into the solution, it reverts to the low-viscosity state. This process is repeatable. To verify the reversibility of the SDS–PEI–nano-silica mixture, a 100 mL solution containing 0.8% SDS, 0.8% PEI, and 0.1% nano-silica was prepared. CO_2_ and N_2_ were alternately injected into the solution at a constant rate of 0.1 L·min^−1^.

**Figure 4:**
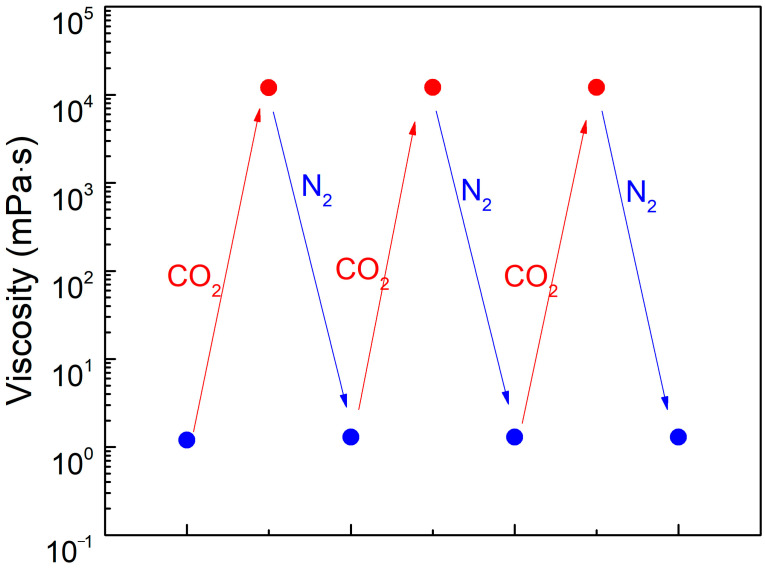
The viscosity changes of SDS–PEI–silica solution in the process of alternately injecting CO_2_ and N_2_. Temperature, 90 °C; salinity, 5000 mg/L; shear rate, 10 s^−1^.

The viscosity changes shown in Figure 4 demonstrate that the viscosity of the SDS–PEI–nano-silica solution increases continuously during CO_2_ injection. The solution transitions from a low-viscosity state, resembling an aqueous solution, to a high-viscosity viscoelastic fluid state, as shown in Figure 4. When the viscosity reaches its maximum value of 12,110 mPa·s, N_2_ is introduced, causing the viscosity to gradually decrease until it returns to its initial level (1.3 mPa·s). The transition in the solution state is attributed to the reduction in the pH of the solution upon CO_2_ injection, which protonates PEI and enhances its interaction with SDS. This process leads to the self-assembly of pseudo-hydrophobic associative polymers, resulting in an increase in viscosity. Upon N_2_ injection, deprotonation occurs, transforming the pseudo-hydrophobic associative polymers into spherical micelles and reducing the viscosity. This high-to-low viscosity transition is repeatable, demonstrating the reversible responsiveness and its potential for repeated use.

### 3.4. Test of Shearing Recovery

To investigate the shear transport characteristics of micellar aggregates formed in the SDS–PEI–nano-silica mixture following CO_2_ response within reservoirs, a rheometer was employed to measure viscosity changes during shear–rest cycles at a fixed shear rate of 100 s^−1^. The viscosity changes following CO_2_ response were recorded in the rheometer during repeated shear–rest cycles.

**Figure 5:**
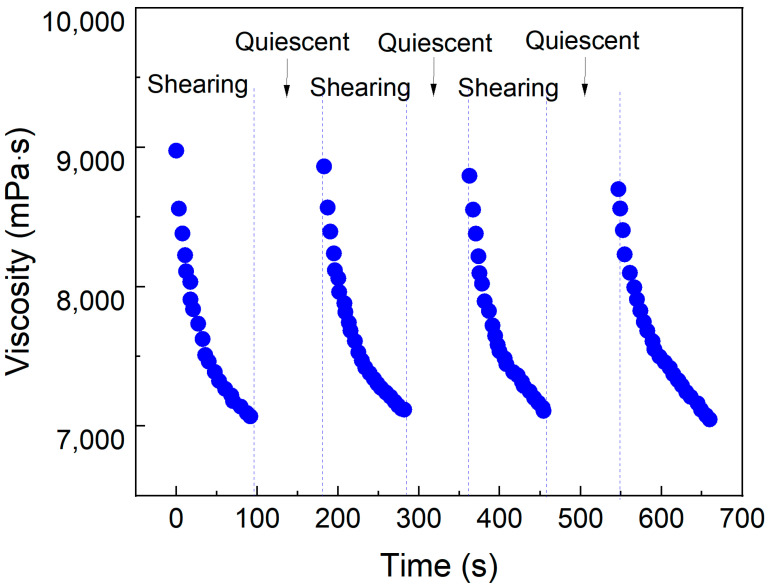
Viscosity changes of SDS–PEI–nano-silica solution during cyclic shear–quiescent states. Temperature, 90 °C; salinity, 5000 mg/L; shear rate, 100 s^−1^.

As shown in Figure 5, the viscosity decreases significantly after shear but rapidly recovers during the rest phase. After multiple shear cycles, the viscosity recovers to a comparable level. Following CO_2_ response, the solution forms a three-dimensional network of entangled pseudo-hydrophobic associative polymers. During shear, the entangled network reorients, while during rest, it recovers. Even after multiple shear cycles, the network remains stable, demonstrating a shear-induced self-healing property. This behavior differs significantly from the shear degradation observed in chemically cross-linked gel, which experiences a notable reduction in strength during deep reservoir plugging. The mixture following CO_2_ response exhibits shear-thinning and rest-induced thickening self-healing properties, facilitating deep placement within reservoirs. This behavior effectively enhances the balanced sweep control capability of CO_2_ flooding in heterogeneous reservoirs.

### 3.5. Thermal Stability and Aging Resistance

To measure the long-term thermal stability of the SDS–PEI–nano-silica mixture, a solution containing 0.8% SDS, 0.8% PEI, and 0.1% nano-silica prepared with salty water was exposed to CO_2_. Following the CO_2_ response, the resulting viscoelastic fluid was sealed in ampoules and placed in an aging tank for long-term curing at 90 °C in a constant-temperature oven. The viscosity of the solution following the initial CO_2_ response was measured after different aging durations. Subsequently, CO_2_ was reintroduced into the aged mixture, and the viscosity was measured again after complete gel formation.

**Figure 6:**
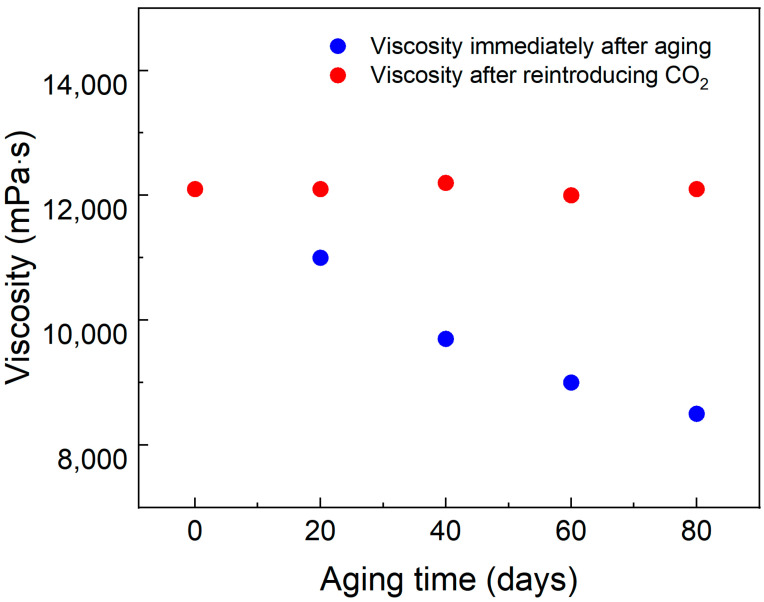
The viscosity of the solution at different aging stages and after re-response. Temperature, 90 °C; salinity, 5000 mg/L; shear rate, 10 s^−1^.

As illustrated in Figure 6, the viscosity of the initially CO_2_-responsive solution gradually decreased after aging for various durations. This behavior may be attributed to the gradual escape of CO_2_ from the solution during prolonged high-temperature aging, which reduces the number of protonated amine groups and loosens the micellar structure. However, after reintroducing CO_2_, the viscosity was restored to its initial level, indicating that the solution maintains long-term stability under high-temperature conditions in a CO_2_-rich environment.

### 3.6. Effect of Salinity

To examine the influence of salinity on the viscosity of the system, brine solutions with salinity levels ranging from 0 to 20,000 mg·L^−1^ were prepared using NaCl. These brine solutions were subsequently used to prepare system solutions containing 0.8% SDS, 0.8% PEI, and 0.1% silica by mass fraction. CO_2_ was then introduced into the solutions until complete gelation occurred, after which the viscosity of the solutions was measured.

**Figure 7:**
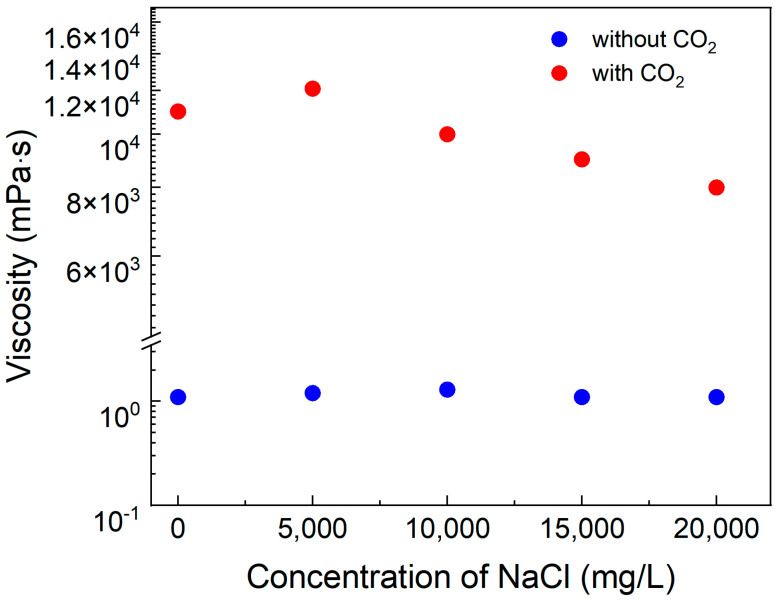
Effect of salinity on viscosity of solution with 0.4% SDS–0.4% PEI–0.1% nano-silica. Temperature, 90 °C; shear rate, 10 s^−1^.

As illustrated in Figure 7, the viscosity did not decrease much with increasing ionic salinity. This phenomenon may be attributed to interactions between ions and surfactant molecules, which influence intermolecular attraction forces. As ionic strength increases, electrostatic repulsion between surfactant molecules is reduced, thereby maintaining interactions between the polymer and surfactants. This results in changes to the viscosity and viscoelastic properties of the solution. The results demonstrate that the solutions maintain stable viscosity at a salinity level of approximately 20,000 mg·L^−1^.

### 3.7. CO_2_ Plugging Test

**Figure 8:**
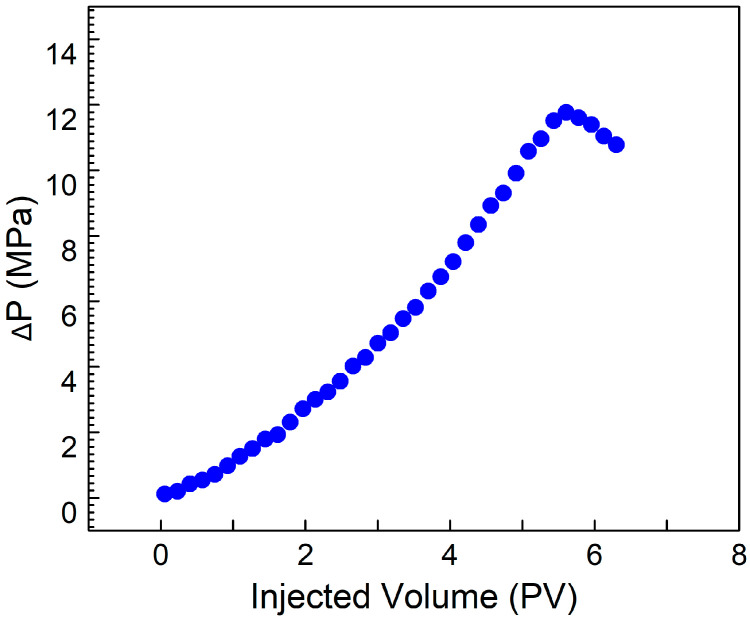
The relationship between ΔP and CO_2_ PV after plugging with 0.4% SDS–0.4% PEI–0.1% nano-silica. Temperature, 90 °C; salinity, 5000 mg/L.

Artificial cores, with gas permeability values of approximately 100 mD and 500 mD, were chosen for plugging experiments employing a solution containing 0.4% SDS, 0.4% PEI, and 0.1% nano-silica. The solution demonstrated a plugging efficiency of over 95% in the cores. Figure 8 shows the plugging solution achieved a breakthrough pressure difference of 12 MPa, highlighting its high performance. This indicates that the solution reacts with CO_2_ within the cores, achieving stimulus-responsive viscosity enhancement. After plugging, the permeability of the cores decreased to below 0.2 mD, which falls into the category of ultra-low permeability. The results of the plugging experiments demonstrate that the solution exhibits excellent plugging performance for cores with permeability of 100 mD and 500 mD, enabling effective sealing of high-permeability zones in formations. This has practical significance for addressing the issue of gas channeling during CO_2_ flooding.

## 4. Conclusions

This study developed a CO_2_-responsive plugging gel composed of PEI, SDS, and nano-silica to solve the issue of gas channeling in the duration of CO_2_ flooding in heterogeneous reservoirs. The findings demonstrated that the help of nano-silica significantly enhances the viscoelasticity of the solution, with the selected formulation identified as 0.8% SDS, 0.8% PEI, and 0.1% nano-silica. The gel exhibited excellent CO_2_-responsive thickening behavior, achieving a viscosity of over 12,000 mPa·s under selected conditions. The CO_2_-responsive gel showed remarkable reversibility, transitioning between high-viscosity and low-viscosity states upon alternating injection of CO_2_ and N_2_. It also demonstrated excellent shear-thinning and self-healing properties, ensuring stability and effectiveness during deep reservoir placement. Furthermore, the gel maintained its performance under high-salinity and high-temperature conditions, with viscosity recovery observed even after prolonged aging. Plugging experiments confirmed that the gel effectively reduced the permeability of artificial cores with gas permeabilities of 100 mD and 500 mD to below 0.2 mD, achieving a plugging efficiency of over 95%. These results highlight the potential of the CO_2_-responsive plugging gel to prevent gas channeling and increase oil recovery in heterogeneous reservoirs, providing a practical and environmentally friendly solution for CO_2_ flooding applications.

## Data Availability

The original contributions presented in this study are included in the article/Supplementary Materials. Further inquiries can be directed to the corresponding author.

## Supplementary Materials

The following supporting information can be downloaded at https://www.mdpi.com/article/10.3390/polym17060706/s1: Figure S1:Viscosity of 0.4% SDS-0.4% PEI with varying nano-silica content in the presence and absence of CO_2_ at 90 °C; Figure S2:Viscosity of 0.4% SDS-0.4% PEI with varying nano-silica content in the presence and absence of CO_2_ at 90 °C.
