# Supplementary material for: CO2-Responsive Plugging Gel with Sodium Dodecyl Sulfate, Polyethyleneimine, and Silica
Source: Polymers (Basel). 2025 Mar 7;17(6):706. doi: 10.3390/polym17060706 (PMC11944695; doi:10.3390/polym17060706)
Supplement: Supplementary file 1 [file polymers-17-00706-s001.zip › polymers-3471318-supplementary.pdf]

Supporting Information for

# $CO_2$-Responsive Plugging Gel with Sodium Dodecyl Sulfate, Polyethyleneimine and Silica

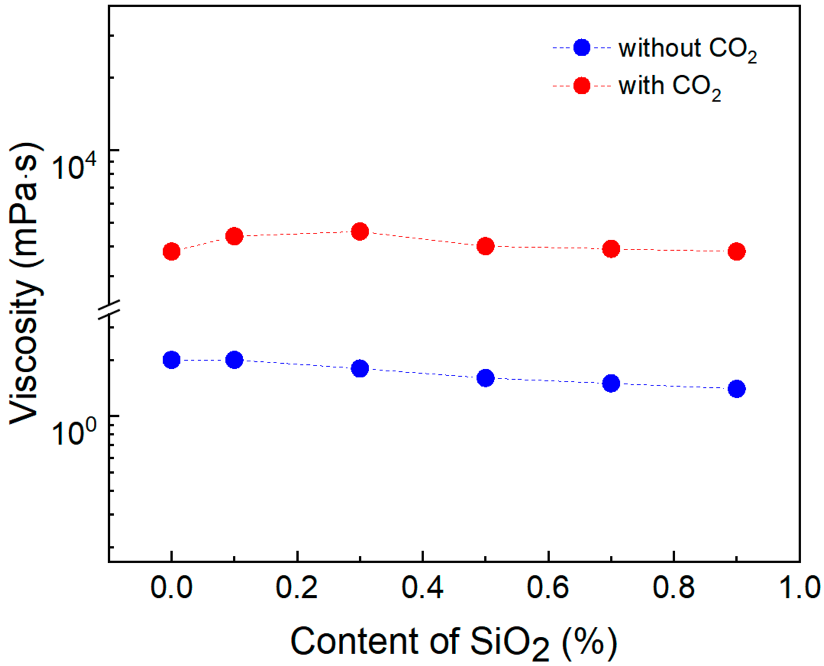


**Figure S1.** The effect of nano-silica content for 0.4% SDS-0.4%PEI on viscosity was studied under conditions of 90°C, a salinity of 5000 mg/L, and a shear rate of 10 $s^{-1}$. The blue points indicate the viscosity measured after $CO_2$ injection, whereas the red points represent the viscosity measured before $CO_2$ injection.

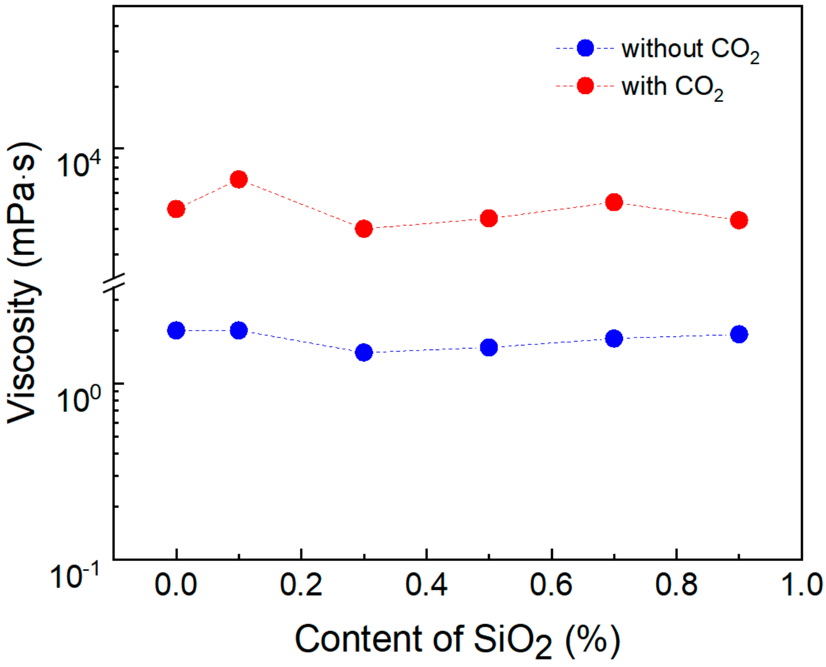

**Figure S2.** The effect of nano-silica content for 0.6% SDS-0.6%PEI on viscosity was studied under conditions of 90°C, a salinity of 5000 mg/L, and a shear rate of 10 $s^{-1}$. The blue points indicate the viscosity measured after $CO_2$ injection, whereas the red points represent the viscosity measured before $CO_2$ injection.
